# Nursing professor as being-cared for in the professor-student relationship

**DOI:** 10.1590/1980-220X-REEUSP-2021-0345

**Published:** 2022-02-28

**Authors:** Raúl Fernando Guerrero-Castañeda, Grever María Ávila Sansores, Sergio Albañil-Delgado

**Affiliations:** 1 Universidad de Guanajuato, Campus Celaya-Salvatierra, Celaya, Guanajuato, Mexico.; 2 Universidad de Guanajuato, Campus Irapuato-Salamanca, Departamento de Enfermería y Obstetricia, Irapuato, Guanajuato, Mexico.

**Keywords:** Faculty, Students, Education, Nursing, Nursing, Qualitative Research, Docentes, Estudantes, Educação em Enfermagem, Enfermagem, Pesquisa Qualitativa, Docentes, Estudiantes, Educación en Enfermería, Enfermería, Investigación Cualitativa

## Abstract

**Objective:**

to understand the meaning of professor as being-cared for that is manifested in the nursing professor-student relationship.

**Method:**

a qualitative phenomenological study, using Martin Heidegger’s philosophical framework. It was carried out at a public university in Mexico. The phenomenological interview was used as a data collection method. An ontic analysis and a second analysis were carried out through Martin Heidegger’s Hermeneutic circle.

**Results:**

within the interviews, 5 main themes were discovered, which were classified as: *Care of physical needs, occupation as care; Nursing care teaching*; *Monitoring and coexisting with professors; Encouraging reflection on care towards authenticity; Students as a person, coexistence and existence of care.*

**Conclusion:**

nursing professors, in addition to being the one who provides students with necessary knowledge for training nursing professionals, share aspects that involve more than science itself. Professors become a collaborator who hopes to contribute to nursing students’ deepest aspects, impacting their academic training favorably.

## RESUMEN

**Objetivo:**

comprender el significado del profesor como ser-cuidado que se manifiesta en la relación profesor-estudiante de enfermería.

**Método:**

estudio cualitativo fenomenológico, utilizando a Martin Heidegger como referencial filosófico. Se llevó a cabo en una universidad pública de México. Se utilizó la entrevista fenomenológica como método de recolección de datos. Se realizaron un análisis óntico y un segundo análisis a través del círculo Hermenéutico de Martin Heidegger.

**Resultados:**

dentro de las entrevistas, se realizó el hallazgo de 5 temas principales, los cuales se clasificaron en: *Cuidado de las necesidades físicas, la ocupación como cuidado*; *Enseñanza del cuidado de enfermería*; *Acompañamiento, coestar del profesor*; *Fomentar la reflexión del cuidado hacia la autenticidad*; y *El estudiante como persona, el coexistir y la existencia del cuidado*.

**Conclusión:**

el profesor de enfermería, además de ser quien provee a los estudiantes con el conocimiento necesario para la formación de profesionales de enfermería, comparte aspectos que involucran más que la propia ciencia. El docente se convierte en un colaborador que espera aportar en los aspectos más profundos del estudiante enfermería, impactando en su formación académica de forma favorable.

## RESUMO

**Objetivo:**

compreender o significado do professor como ser-cuidado que se manifesta na relação professor-enfermeiro-aluno.

**Método:**

estudo fenomenológico qualitativo, usando o referencial filosófico de Martin Heidegger. Foi realizado em uma universidade pública no México. A entrevista fenomenológica foi utilizada como método de coleta de dados. Foi realizada uma análise ôntica e uma segunda análise, através do círculo hermenêutico de Martin Heidegger.

**Resultados:**

dentro das entrevistas, foi feita a descoberta de 5 temas principais, que foram classificados como: *Cuidar das necessidades físicas, ocupação como cuidado; Ensino do cuidado de enfermagem; Acompanhamento, coexistência do professor; Incentivar a reflexão do cuidado para a autenticidade; e O aluno como pessoa, a convivência e a existência do cuidado*.

**Conclusão:**

o docente de enfermagem, além de ser aquele que proporciona aos alunos os conhecimentos necessários para a formação dos profissionais de enfermagem, compartilha aspectos que envolvem mais do que a própria ciência. O professor torna-se um colaborador que espera contribuir com os aspectos mais profundos do estudante de enfermagem, impactando de forma favorável em sua formação acadêmica.

## INTRODUCTION

Teaching is a complex action, a form of relationship in which there is interaction between professors and students. Nursing professors intend to act as beings that are oriented to support students in their training process and that, through the use of different teaching methods and strategies, can convey nursing knowledge. However, this communication occurs in a dialogical pedagogical relationship, where other tools are used that allow students to fulfill and develop optimally beyond complying with a graduation profile, as a person of care itself^(1)^.

On the other hand, it is necessary to highlight that future nursing professionals are able to understand what nursing care implies^(2)^. This complex understanding goes beyond empty knowledge, it also contemplates not neglecting to have skills, competencies and abilities required to deal with various care situations. Nursing pedagogy could focus on teaching humanized care^(3)^ that allows training professionals of integrity capable of seeing the other as a person who is cared for, not just as a patient suffering from a health problem.

Nurses in training are expected to seek to provide care that is appropriate for each person cared for^(4)^, understanding that each individual is a unique and unrepeatable being. In this way, a humanized care is consolidated. Watson mentions that this care activity is comprised of two main elements, a caregiver and a being that is cared for^(5)^, which, while this care interaction develops, a bond is formed between both people, thus living a moment of care.

The classroom becomes a space where this teaching-learning process takes place and where there are two main actors in this process (professor and student)^(6)^. As a result, a relationship is obtained where both collaborate in the teaching-learning process^(7)^, favoring the creation of a link where professors, beyond imparting theoretical knowledge, could become a substantial element of care itself.

Teaching how to care is monitored by experiences that highlight the positive acts of nursing that can impact training^(8)^, hence the relevance of addressing the professor-student relationship from a care perspective. Various studies address precisely the importance of care being conveyed through caring for others. On the one hand, professors’ behavior should inspire human care itself^(9)^ and relational communication as training for care and nursing students’ experience, on the other, faculty members are subjective and nursing students recover the experience of care through various subjective elements that occur in a pedagogical relationship^(10)^.

With these first reflections and given that it has been shown that, in the nursing professor-student pedagogical relationship, professors, in order to teach how to care, they must be aware of their teaching action from the perspective of care and can become caring beings. Nursing professors live this care in the classroom, from a phenomenological perspective. Professors, to convey care, would be understood as a being-cared for where they can shape the experience of caring for students and promote care^(11)^.

For this reason, the daily life of professors’ teaching process can reveal, on the one hand, the meaning of care and, on the other, the way in which professors are understood as being-cared for and the impact that this has on students’ education, so it becomes a phenomenological encounter. Therefore, the question arose: what is the meaning of professors as being-cared for that is manifested in the nursing professor-student relationship?

Starting then from the fact that the professor-student pedagogical relationship generates caring relationships in coexistence, this study sought that meaning from professors’ daily life^(12)^, constituting nursing professors as beings-cared for from their own experience, in such a way that their being understood in the world of care can be addressed, using Martin Heidegger’s phenomenological hermeneutical philosophical and methodological framework, which constitutes an existential concern of being in the world^(12)^. The objective was to understand the meaning of professors as being-cared for that is manifested in the nursing professor-student relationship.

## METHOD

### Design of Study

This is a qualitative phenomenological study based on Martin Heidegger’s philosophical framework, conceiving the professor-student relationship as a care relationship that unfolds in space and how it interacts in time with the actors involved.

### Population

Professors from two campuses of a public university participated; these professors were nurses by training and taught classes on disciplinary subjects. This group was accessed through an email invitation addressed to all nursing professors from both campuses. This email specified the research’s title, purpose, the selection criteria and the procedure, in addition to the contact information. Those who contacted the researchers expressing their desire to participate were selected.

### Scenario

The research was developed in two campuses of a public university in the state of Guanajuato, Mexico, from March 2019 to December 2020.

### Selection Criteria

The selection criteria for the participation of professors were having a degree in nursing, being a nursing professor, a minimum of 3 years of teaching experience teaching practical or theoretical nursing subjects.

### Sample Definition

A convenience sampling was carried out, given that people from teaching contexts where there was a nursing professor- student relationship were consulted. This criterion was determined based on the selection criteria, also considering Heidegger’s phenomenology. Professors’ understanding, in their very being, was attended to and they were the ones who identified themselves with the purpose of the research and were also available to participate. The invitation was opened to the two campuses’ professors. The professors’ answers were received at the same time that invitations were continued to be sent by email. When saturation was reached, the invitations were suspended, and this happened when the information became redundant in initial analysis, obtaining a sample of 10 professors.

### Data Collection

Phenomenological interviews^(13)^ composed of two trigger questions were conducted: what does care in the professor- student relationship mean to you? How do you take care of your nursing students? The interview allowed collecting the information provided, in order to reveal the phenomenon that was the object of study. The interviews were conducted by the main researcher, who is an expert in phenomenology and his method, as well as interviews with this approach. One more participated as a listener (because he was a researcher in training) before participants’ acceptance, so it was done in this way, since it was not mandatory. Participants showed no discomfort from this. Five of the ten interviews were conducted in this way and the main researcher conducted the full interviews.

### Data Analysis and Treatment

The analysis process was carried out in matrices in M. Word 2018^®^. Information analysis was carried out through Martin Heidegger’s Hermeneutic circle^(12)^, which consists of 3 moments: (1) Pre-understanding, highlighting the characteristics that include professors in the classroom and the way in which they care for nursing students. It comprises the identification of units of meaning in a matrix; (2) Understanding, designing the existing essential representations around the care performed by nursing professors. This moment is carried out in two matrices, one being to group the units of meaning looking for convergences between them; and (3) Interpretation, conveying the phenomenon according to characteristics and the way it is developed. Care reveals the meaning it has for each professor. This moment is carried out in a third matrix where grouped units are interpreted based on existing literature in related studies and Martin Heidegger’s concepts, mainly from his work *Being and Time*. The analysis was carried out by three researchers independently and, later, the units of meaning and themes were unified by consensus among researchers. The results were presented to participants, to validate the context of the units of meaning.

### Ethical Aspects

The project was approved by the Research Committee, with code DCSI-CI201920408-7, and by the Bioethics Committee, with registration CBCCS-02327052019, both belonging to the *Universidad de Guanajuato*. The General Health Law in Health Research Matters was respected. Participants signed the Informed Consent Form, to comply with confidentiality. A code was assigned to each participant at the time of being audio recorded, for subsequent transcription and analysis. Scientific rigor criteria were used for qualitative research of confirmability, transferability, value of truth and theoretical- epistemological coherence.

## RESULTS

A total of 10 interviews were conducted, with an average duration of 44.15 minutes, ranging from 30 to 65 minutes. Regarding participants’ characteristics, the average age was 43.14 years (min 29, max 56). The average number of years of teaching was 14.42 years (min 4, max 32). All participants are women, 8 have a master’s degree and 2 have a doctoral degree, 7 of them report using a pedagogical and/or disciplinary theory in their teaching, while 3 report not applying either of the two. As a result of the interviews, five main themes could be revealed, namely: *Care of physical needs, occupation as care; Nursing care teaching*; *Monitoring and coexisting with professors; Encouraging reflection on care towards authenticity; Students as a person, coexistence and existence of care*.

### Care of Physical Needs, Occupation as Care

In this section, it was possible to group the importance for nursing professors caring for students’ physical needs and the importance for them of promoting that care:

*I am always trying to get them to be satisfied first as subjects. If they are not well, it will be very hard for them to be well with their environment (P1).*

*They do not have to ask permission to go to the bathroom, they can go, and if they are hungry or that, well, we will accommodate according to the activity that we are doing, they can eat (P4).*

Professors state that future nursing professionals must be able to provide care for themselves so that they can provide it to other people and make it easier for them to identify each student’s needs. Care with food, rest, elimination and overall health is highlighted:

*Definitely, first you need to be well and master many things, not only the nursing area, but also learn to control all those emotions that we experience (P3).*

*First, you take care of yourself and love yourself, then you care for a patient. If you don’t take care of yourself and if you don’t love yourself, how are you going to meet patients’ needs? (P9).*

### Nursing Care Teaching

In this topic, the need of professors to take care of the environment within the classroom is mentioned, seeking that students can function authentically as the main objective. The importance of taking care of the environment within the classroom is mentioned, as well as looking for strategies that favor learning and catching students’ attention. Professors report:

*Also, like caring for their environment where they are going to perform their tasks (P6).*

*I’m working on that understanding that they learn differently… I’m trying to generate instruments, generate resources that are more visual or more attractive to them (P8).*

*I observe those who need to be more attentive and follow them, and then I like to observe first (P9).*

Nursing professors aim to establish a learning environment that allows students to develop within a classroom in which values are encouraged. On the other hand, professors seek the collaboration of different characters within the classroom that favors learning. Professors report:

*I have always told them to try to work as a team… sometimes it happens to me that they don’t work and I change teams (P1).*

*From the first day I ask you, what are their names? But above all, how do you prefer to be called?… to touch on these topics that allows us to reinforce in them many attitudes (P5).*

*I tell him that it is your life project that you are doing based on other people, they are telling you at the end of the day I am working on what I like (P7).*

### Monitoring and Coexisting with Professors

In this topic, the need of professors to monitor students’ learning process is mentioned, in which it is allowed to make interventions within students’ academic and also personal situations. There is a concern of professors for aspects that go beyond academic situations. Participants express:

*Sometimes they come to talk to me about situations… I think this is a form of reward, because I care a little about them (P1).*

*I have been touched by situations in which students approach me as if I were their tutor (P7).*

*Monitoring is simply being there with them, not necessarily physically, but maybe with a little message, via email, letting them know that someone is listening to them and is there for them (P10).*

### Encouraging Reflection on Care Towards Authenticity

Within this topic, it is found that nursing professors intend that students make a reflection on what nursing care implies. To this end, professors, within their classes, have the opportunity to work with real cases that are related to the hospital environment. This is stated by participants:

*When you go to work, they don’t ask you if you get along or not with the partner, you are hired and you have to fulfill it, so here you fulfill it (P2).*

*There’s a point that we don’t know if we remember in the evaluation that says if you put practical examples, for example, from everyday life for them to identify (P5).*

Moreover, the existential gap between the knowledge acquired within the classroom with the problems that arise when students face professional care situations is emphasized. This was stated by the following participants:

*For me, this is also taking care of them, preparing them to face a work environment… saying that they will have to face these situations (P3).*

*You will not treat someone as badly as they treated you, and so that you will always remember that one day you were a student and that one day you will see your colleagues and see that they have the same needs as you did (P9).*

### Students as a Person, Coexistence and Existence of Care

Nursing professors, in addition to seeing students as a person with whom they must share their knowledge, they see them as persons who have needs that are beyond the academic field. That is why professors consider it necessary to provide support and monitoring. It is when professors are conceived as beings who takes care of students in the daily academic relationship. Participants report:

*For me, as a professor, I have to understand that students are still a person, that they need respect, recognition, to have their needs met and that they need to connect with me (P3).*

*For me it is important from the beginning to recognize them as people and treat them as people, for me it is important to get to know them, understand their way of expressing themselves, understand their personal situation (P5).*

*As a person… who has feelings, who has health, who has a family, so take care that they have a balance of all these aspects (P6).*

*As I am in this flexible part and I understand that they are also people, not only do I see them as students, I understand that after here they have a house, maybe with several problems… you should take care of the other for me from that moment on (P10).*

## DISCUSSION

The professors had an average of 14.42 years of teaching. This coincides with some studies where the participating professors have between 1 and 18 years of teaching^(8,14)^. They were all women, which may be related to nursing training, which continues to be predominantly female^(14)^. Most have a master’s degree and apply some pedagogical or disciplinary theory. In this sense, some studies refer to the importance of professor training that gives them disciplinary experience^(15)^, as well as the importance of mastering pedagogical concepts^(16)^, since professors, based on teaching experience, their disciplinary training and pedagogical mastery, could contribute with training oriented towards innovative teaching-learning paradigms^(10,17)^.

Professors, in the pedagogical relationship with students, understand that they have basic needs, including physical ones. This can be seen in the speeches where they point out dealing with these physical needs, such as food, rest, fulfilling these needs, with the purpose of promoting self-care for daily needs, to be able to interact in a better way in the classes. On the one hand, the promotion and care of these needs and counseling on healthy habits stand out.

In turn, the literature highlights that it is necessary to promote self-care in students^(8)^. It has been pointed out that, during undergraduate study, nursing students have a poor sleep pattern, high level of stress and an unhealthy diet^(18)^. Professors become promoters of students’ own care, meeting their needs in various aspects of life and living them on a daily basis.

Professors become involved in the lifestyle that students lead through different actions that revolve around the benefit of caring for them^(19)^. This is why they tend to find the need to take care of students and promote behaviors that favor their health status that allows better academic performance^(20)^. They are aware of the impact that it can cause in nursing students’ lives, by allowing them to experience care in its most daily forms, and occupation is essential. For Heidegger, there are two ways of living through praxis for human beings: the first is when they are immersed in daily activities without noticing their own existence; the second is when the person is aware of their existence, therefore, that awareness is what can favor that care towards themselves and towards others^(12)^.

The pedagogical dynamic is usually abandoned in everyday life^(12)^, and professors contemplate this dynamic to make care possible in a first approach to the occupation of personal care. Thus, everyday care becomes not just an action to be conveyed, but an act that must be contemplated and lived even in aspects as simple as meeting students’ needs beyond the academic scope.

Professors live in the personal care dynamics, monitoring in addition to other needs, not only physical ones, but orientation and monitoring needs, referring to it as similar to tutoring, but that coexist with students in their teaching practice. These are usually academic needs and also personal in aspects that stand out from the classroom.

Thus, they have a notable and positive influence, since care is given in the relationships between people who live together and understand each other beyond their superficiality^(21)^. They are aware that monitoring is essential in training and also that they cannot improvise it^(22)^. This is vital, since they intend to collaborate with students in monitoring them outside the classroom in the sense of being-there present for them.

Coexistence and co-existence are essential to reveal the essence of caring for the self^(12)^, which is why they mention the importance of monitoring in resolving personal situations and that this satisfies part of future nursing professionals’ training process. Phenomenologically, it is constructed that professors are open in co-existing with students and their essence of being is manifested in coexistence with the other, understanding it in its dimension of being-there^(12)^, since students are also thrown into the pedagogical dynamics. The coexistence of both builds the meaning of care by integrating this care in various daily situations, both coexist in the world of life^(23)^. When contemplating all forms of care, this can mean the experience itself and contribute to the formation in a sense of doing for the other.

Professors are understood in the pedagogical relationship as caring beings who experience concern or request^(12)^, since they realize in these basic daily forms that this is not enough. On the one hand, they contemplate aspects outside the classroom and, on the other, they recognize that traditional teaching needs to change to make teaching care something deeper in students.

If this relationship of coexistence is contemplated phenomenologically, then, open to the possibilities of care, the meaning of the pedagogical relationship is constructed as a request or concern. Thus, instead of depriving students of the possibility to practice and live their care, professors anticipate giving back the care, in ways that they can live it^(12)^, motivating the freedom to experience care in the classroom.

They highlighted that they seek different strategies that allow students to obtain learning that is more meaningful and interactive. Therefore, they use different activities monitored by dynamism, seeking to catch students’ attention. These activities range from games, which promote learning, to interactive resources.

The importance of actions by nursing professors that allow creating an environment based on trust and tranquility for students^(15)^ has been evidenced, which is why it is allowed to carry out interventions within the classroom that lead to this result. In addition to this, didactic techniques are used that favor the interest within the class by students^(14)^. This allows students to carry out different types of learning, such as self-learning, interactive learning^(24)^ and collaborative learning. Therefore, they intend to keep students motivated^(25)^, by encouraging them to follow and seek the optimal conditions to be able to develop nursing, mentioning the limits that exist and the objective to which they can aspire.

Professors maintains this coexistence with other beings, allowing the continuous reflection of the care process in the promotion of this coexistence as a sense of being themselves. Phenomenologically, it constructs the meaning of itself as care in didactic actions, it becomes a Dasein of care^(12)^. The goal is for students to live care, thus care becomes a Hermeneutic task^(26)^, then, through the above, it is understood, interpreted and applied in everyday life.

When the didactic strategies are dynamized, it is allowed and opens to the possibilities that allow, not only to memorize the contents, but to experience them. Professors encourages reflection on care, from examples from their own practice of nursing care, which they replicate in their classes, to examples they design, in order to help students think about this care. The findings converge on the importance of not only conveying theoretical knowledge, but also living it with examples.

Showing students the different situations that they can face favors critical thinking and reflection. In this way, the care given by students is a reflection of what professors have fostered^(19)^. Furthermore, by incorporating the reflective practice of the contents, the link between theory and practice is favored, reflective classrooms are encouraged and trust is generated in the pedagogical relationship^(8)^. On the other hand, the care relationship becomes more human^(16)^.

It is interpreted that, in the ways in which professors move in their being of care and in the relationship of coexistence with students, care is developed as an act of interaction, they are contemplated as being-there willing to seek the sense of being of students^(12)^ and the possibility of living care becomes tangible in each action.

Professors take care of students, not superficially, but they encourage reflection to move students; in this way, in addition to theoretical teaching, they understand that care is conveyed from the simplest forms to the most complex, so they use reflection to help students not to experience care in inauthenticity^(12)^.

In this reflective search, it is contemplated that inauthenticity would be a form of pedagogical relationship barely focused on conveying knowledge; however, they conceive that it must motivate the student-being to search for authenticity, for a dynamic, reflective, critical care lived day by day, which will allow them to reflect on their professional practice.

Professors see students as a being-there, a person who is not only there to absorb knowledge, who would otherwise live in inauthenticity, but they recognize that they seek authenticity of care. They interpret their self-care as an understanding of students as a person with needs, with dignity, as a reflective person, who contemplate basic physical needs, personal emotional and interrelationship situations that contribute to their training in care. The coexistence between both favors this understanding^(12)^.

Thus, it has been found that students’ training must be framed in a relationship that allows students to value themselves as a person, so they must recognize themselves as a person to recognize the people they care for^(1)^. Care is given between people who recognize themselves in a network of values, beliefs and modes of motivation^(27)^. Care, then, is essential from the point of view of pedagogical relationship that will be reflected in the people cared for.

Hermeneutically, care is interpreted in this relationship and applied in the most sublime ways^(26)^. The intersubjective relationship between professor and student in search of understanding care signifies this as an act of authenticity that goes beyond the content conveyed, allowing students to make their own decisions in relation to academics, as well as in their personal life; in this way, they become a collaborator in the search for the essence of care as a future nurse.

It is favorable for students to develop a bond of trust with professors, which is based on humanized care that allows concern and affection to develop in this relationship, developing active listening^(17)^, monitoring and the need to care for the other allowing reciprocity.

Professors take care of students to make them authentic, a caring being, therefore, helping students to seek that sense of being a nurse from their training. Thus, care lives, because it not only refers to teaching content, but to living care in all its forms inside and outside the classroom.

Phenomenologically, care becomes an action of doing with the other, for the other and by the other. Professors is meant as being-cared for in the relationship with students, where the existence of the act of caring is lived in aspects that are so essential for the understanding of being.

Learning to care requires an integration of content with experiences in the classroom that are transformed by students^(28)^. It occurs in a dialogic relationship between professor and student seeking to transform that knowledge into a lived reality^(29)^. Care becomes hermeneutically an existential totality that, although it is not a sum of elements, is an authentic articulation that allows it to be lived as a transforming experience^(12)^.

In essence, professors understand each other in an authenticity that is capable of moving and transforming students as beings of care^(29)^ as long as they reflect and live teaching day by day as a binding and reflective process. As long as both beings understand each other in authenticity, the meaning of being-cared for is manifested, since professors in this relationship become free to help the other in the form of occupation, concern and request^(12)^.

The care in their being-professor involves in these forms of daily manifestation the care of themselves and towards others. Care becomes manifest in the co-existence and coexistence of both^(12)^ and gives sense and meaning to the existence of this care in the pedagogical relationship where both are transformed. This allows revealing that the professors interviewed converge in the attributed meaning endowed with elements whose ontology confers that sense of care beyond the theory conveyed.

The contributions of this study for teaching nursing show the importance of understanding nursing care as the very essence of the human being as a being of care thrown into everyday life. Care becomes lived and can be manifested in personal care and above all in the care of the other, with the other and for the other, in order to ensure that students are not inauthentic, but internalize the complexity of caring with a comprehensive approach in daily classroom activities, personal relationships with other beings, caring for themselves and others.

This study presented limitations, one of them was the extension of time set, since it was difficult to agree on the interviews and it was necessary to move them on some occasions, due to academic load. Similarly, it represents an opportunity to address other forms of care based on this phenomenon. On the other hand, the results were quite extensive, so the most relevant ones were selected for this article. The limitations associated with the method are that the results cannot be generalized, but they do represent a reflection of what professors are and do, on a daily basis, for students and with them, to live the teaching of care.

## CONCLUSION

Nursing professors, in addition to being the ones who favor conveying pertinent knowledge for training future nursing professionals, convey cognitive aspects that go beyond nursing theory. Professors actively participate in different aspects of students, seeking their well-being and understanding; on the other hand, they seek to adopt an environment within the classroom which is pertinent to develop learning and which also collaborates with the innovation of techniques that allow students to acquire knowledge and that this in turn causes a greater impact on their life.

In the same way, professors seek to collaborate in the resolution of problems that students may face. To this end, they are open with students, allowing an approach in which active listening is developed. They seek to collaborate with students in favor of their well-being, sometimes allowing themselves to make references with different professionals that allow the resolution of the problems that have arisen.

Finally, nursing professors are allowed to bring students closer to challenges with which they can face in becoming a nurse and introduce them to the work environment, in addition to inviting them to develop fully, by performing the function of being a support for students at a time.

## ASSOCIATE EDITOR

Cristina Lavareda Baixinho
